# 5-Methylheptadecane: Sex Pheromone of the Broom Twig Miner, *Leucoptera Spartifoliella*, a Biological Control Agent for the Scotch Broom

**DOI:** 10.1007/s10886-023-01446-x

**Published:** 2023-07-21

**Authors:** Ashraf M. El-Sayed, Barry Bunn

**Affiliations:** 1 Canterbury Agriculture & Science Centre, The New Zealand Institute for Plant and Food Research Limited, 74 Gerald St, Lincoln, 7608 New Zealand; 2 The New Zealand Institute for Plant and Food Research Limited, Batchelar Road, Palmerston North, 4474 New Zealand

**Keywords:** Broom twig miner, *Leucoptera spartifoliella*, Sex pheromone, Scotch broom, *Cytisus scoparius*

## Abstract

The broom twig miner, *Leucoptera spartifoliella* (Hübner) is a highly specific biological control agent for the Scotch broom, *Cytisus scoparius* (L.). *Cytisus scoparius* has become a major invasive weed spreading in North America, South America, Australia and both the South and North Island of New Zealand, causing a major disturbance to the ecology of the area where it has been established. Currently, there is no tool available to monitor the population density, dispersal, and establishment of *L. spartifoliella*. This work was undertaken to identify the sex pheromone of *L. spartifoliella* and develop a monitoring tool for this biological control agent. Analysis of the extract of the female sex pheromone gland by gas chromatography/mass spectrometry and chemical ionization identified a single compound in the extract. Using the mass spectral data and synthesis of candidate compounds, this compound was identified as 5-methylheptadecane. In a field trapping trial, testing various doses (0.01, 0.1 and 1 mg) of 5-methylheptadecane and virgin females as a positive control, the highest male catch obtained was in traps baited with the 1 mg dose. When testing two releasing substrates for the pheromone (i.e. a glass vial containing a cotton ball and another containing white septum), the male catch in a trap baited with a glass vial with the cotton ball was significantly higher than in a trap baited with a white septum. The identification of the sex pheromone of *L. spartifoliella* will help to detect and determine population densities, distribution, and dispersal of *L. spartifoliella*.

**Supplementary Information:**

The online version contains supplementary material available at 10.1007/s10886-023-01446-x.

## Introduction

The Scotch broom, *Cytisus scoparius* (L.) is a perennial leguminous shrub native to western and central Europe. *Cytisus scoparius* has spread beyond its native habitats and has become an invasive species and a serious weed in temperate areas of the United States of America (USA), Canada, Hawaii, Chile and Argentina, New Zealand, Australia, India, Iran, Japan and South Africa (Holm et al. 1979; Parsons and Cuthbertson 1992; Hosking et al. 1998; Peterson and Prasad 1998; Isaacson 2000). It was introduced to New Zealand in the late nineteenth century as an ornamental plant, but it is a very aggressive fast-growing weed with the capability to grow by forming dense, impenetrable, monospecific stands that degrade native grasslands, forests, rangelands, and agricultural lands. It prevents the regeneration of natural forests and prairies and creates fire hazards (Syrett et al. 1999). Because of its association with nitrogen-fixing bacteria, it is very competitive in areas with poor soils and can alter the nutrient cycling of invaded areas (Peterson and Prasad 1998). Therefore, it can change the types of plants that can survive where it has been growing, entirely disturbing the ecology of an area where it has become established. The damage includes preventing the seedlings of native species from establishing, changing habitats and other plant species being present which can lead to further weed invasion.

The broom twig miner, *Leucoptera spartifoliella* (Hübner), is a whitish microlepidopteran ca. 3–4 mm long, whose larvae specifically mine the young stems of the Scotch broom, *C. scoparius* (L.) and the Spanish gold broom, *C. purgans* (L.) (Parker 1964). In the 1950s, *L. spartifoliella* was accidentally introduced to New Zealand. Later it was introduced from its native Europe to the USA for the biological control of broom in California in the early 1960s and it was introduced to Australia in 1990 (Coombs et al. 2004). The larvae damage the broom by feeding on stem tissues (Gourlay 2021). In New Zealand, a large outbreak of broom twig miner can occur causing severe damage to broom plantations by reducing their growth and longevity (Gourlay 2021). Also, this insect was found to have a noticeable damaging effect on broom shrubs in North America (Coombs et al. 2004). Therefore, this biological control agent could help to control the spread of this aggressive invasive plant outside its native habitat.

The development of a monitoring system based on traps baited with the sex pheromone of *L. spartifoliella* will help in the detection of newly-established populations in new habitats, determining population densities, distribution, and dispersal of this biocontrol agent. We report here on the identification, synthesis, and behavioral evaluation of the sex pheromone of *L. spartifoliella*.

## Materials and Methods

### Insects

Pupae of *L. spartifoliella* were collected from silk cocoons on the stems of *C. scoparius* during the spring and summer (October/November 2018 and 2019) from a Scotch broom plantation near The New Zealand Institute for Plant and Food Research Limited (Plant & Food Research) Campus, Lincoln, New Zealand. Pupae were housed individually in 30-ml plastic cups (Solo, Mason, MI) with a moist dental roll. All pupae were kept in a controlled-environment room under a 16 h:8 h L:D photoperiod and 23 °C, and newly emerged adults were sexed under the microscope. Virgin females were used in sex pheromone extraction and field trapping experiments as a positive control.

### Pheromone Gland Extraction

The sex pheromone glands of 2- to 3-d-old calling females (*N* = 15) were dissected during the first 2 h of the scotophase, when most of the females were found to call, and extracted in 20 μl of *n*-hexane (Merck Ltd, Darmstadt, Germany) in a 0.5-ml conical vial (Wheaton, Millville, NJ, USA) cooled with liquid nitrogen for 5–10 min. After all glands had been excised, the vial and its contents were brought to room temperature, and the liquid phase was transferred to a 1.1 ml conical glass vial (Alltech, Deerfield, IL, USA) for storage at − 80 °C before analysis. The single compound in gland extract was quantified using an external standard method (Scott 1995). For this, five females were extracted individually in 10 μl of *n*-hexane. Known amounts (0.1, 1.0, 10.0, and 100.0 ng) of pheromone compound were injected into a gas chromatograph/mass spectrometer (GC/MS). The integrated results were subjected to linear regression analysis (SAS Institute Inc. 1998) to determine the relationship between the amount and peak area.

### Gas Chromatography/Mass Spectrometry (GC/MS) Analysis

Gland extracts and synthetic chemicals were analyzed on a Saturn 2200 GC/MS (Varian Inc., now Agilent Technologies, Palo Alto, CA, USA). The ionization voltage was 70 eV (electron impact ionization) with mass scanning from *m/z* 30–500. Three different capillary columns were used in this study: (1) a non-polar 30 m × 0.25 mm ID × 0.5 μm VF5-MS capillary column (Factor four, Varian); (2) a polar 30 m × 0.25 mm ID × 0.5 μm VF23-MS capillary column (Factor Four, Varian); (3) a chiral a 30 m × 0.25 mm i.d. × 0.25 μm CycloSil-β chiral column (J&W Scientific). The temperature of the injector was set to 250 °C. Helium was used as carrier gas at a constant flow rate of 0.8 mL min^− 1^. The transfer line temperature and manifold temperature were set to 250, and 50 °C, respectively. Samples were analysed at two ion trap temperatures (250 and 110 °C). Chemical ionization (CI) with acetonitrile (ACN) as the reagent gas in the GC/MS system was used to determine the molecular weight of the compound present in the sex pheromone gland extract. ACN forms the cation (M + C_2_H_2_N)^+^, resulting in a an ion at *m/z* (M + 40)^+^ especially with saturated alkanes. All CI analyses were conducted with an ion-trap temperature of 110 °C, a manifold temperature of 50 °C, and an ion-source temperature of 160 °C. Data were analyzed using the MS Workstation software (version 6.9.3, Varian). For the three columns, the injection was splitless and the column oven was programmed from 80 °C (held for 1 min) to 240 °C at 10 °C/min^− 1^ and then held for 15 min. The female gland compound was identified by comparing the retention indices and mass spectra with the synthetic compound on the three different columns.

### Synthesis

#### Synthesis of 5-Methylheptadecane

**Fig. 1:**
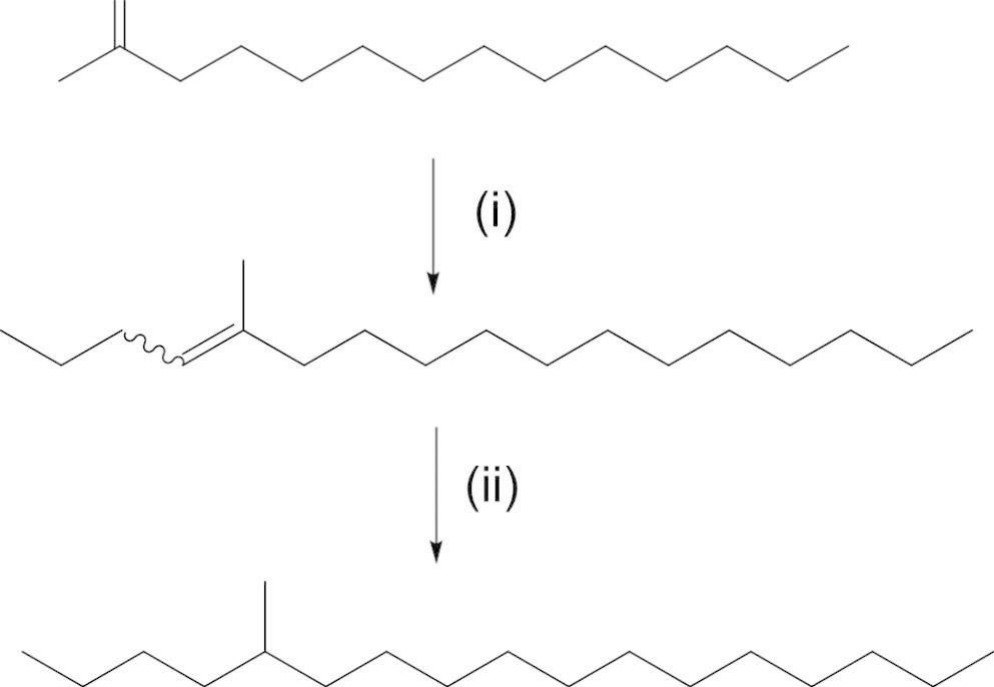
Synthesis of 5-methylheptadecane. Reagents/Conditions: (**i**) nBuLi/butyltriphenyl phosphonium bromide/THF, -20 °C; (**ii**) Pd(C), H_2_, petroleum ether

5-Methylheptadecane was synthesised as shown in Fig. 1. The intermediate alkene was obtained as a mixture of *E*- and *Z*-isomers, which were hydrogenated to give the product as a racemic mixture.

#### Synthesis of (E/Z)-5-Methylheptadec-4-ene

^n^Butyl lithium (0.5 ml, 1 mM, 2.0 M in cyclohexane) was added dropwise over 1 min to a stirred suspension of butyl triphenylphosphonium bromide (398 mg, 1 mM) in 20 ml tetrahydrofuran (THF) at − 20 ^o^C. There was an immediate orange colour. This mixture was stirred at − 20 ^o^C for 20 min when a solution of 2-tetradecanone (212 mg, 1 mM) in THF (3 ml) was added dropwise over 1 min. The reaction was allowed to warm to ambient temperature and stirred for 18 h. NH_4_Cl (10 ml, sat. aq.) was added and the aqueous phase was extracted with diethyl ether (2 × 10 ml). The combined organics were washed with brine (10 ml, sat. aq) and dried with MgSO_4_. After filtration the solvent was removed in vacuo and the crude material was purified using column chromatography to give the alkene as a mixture of *E*- and *Z*-isomers, which were used as a mixture in the next step. Yield = 52 mg, 20%. Purity = 98% by GC/MS. ^1^ H NMR (700 Mhz. CDCl_3_): δ 5.14 (1 H, m, both isomers), 2.20–1.96, (4 H, m, both isomers), 1.60 (3 H, s minor isomer), 1.59 (3 H, s, major isomer),1.40–1.20 (22 H, m, both isomers), 0.92 − 0.85 (2 × 3 H, both isomers). ^13^C NMR (175 MHz. CDCl_3_): δ 135.4, 125.0, 124.31, 124.30, 41.4, 39.7, 31.9, 31.8, 31.6, 30.0, 29.9, 29.72, 29.71, 29.68, 29.67, 29.65, 29.60, 29.3, 29.1, 28.1, 28.0, 27.75, 22.73, 26.9, 23.4, 23.3, 23.1, 22.7, 22.6, 20.7, 18.8, 15.9, 14.4, 14.1, 14.0, 13.9.

#### Synthesis of 5-Methylheptadecane

A mixture of *E-* and *Z-*isomers of 5-methylheptadec-4-ene (50 mg) was hydrogenated in petroleum ether in the presence of 10% Pd (C) catalyst (30 mg). After 90 min the reaction was complete as judged by GC/MS analysis. The catalyst was removed by filtration and the solvent was removed *in vacuo* to give the product as a waxy solid. Yield = 35 mg, 69%. ^1^ H NMR (700 Mhz. CDCl_3_): δ 0.86 (3 H, d, 6.3 Hz), 0.9 (6 H, m), 1.35–1.25 (29 H, br). ^13^C NMR (175 MHz. CDCl_3_): δ 37.1, 36.8, 32.7, 31.9, 30.0, 29.8, 29.67, 29.71, 29.72, 29.75, 29.4, 27.1, 23.1, 22.8, 22.8, 19.7, 14.2, 14.1.

#### Synthesis of 6,10 Dimethylhexadecane and 5,9 Dimethylhexadecane

**Fig. 2:**
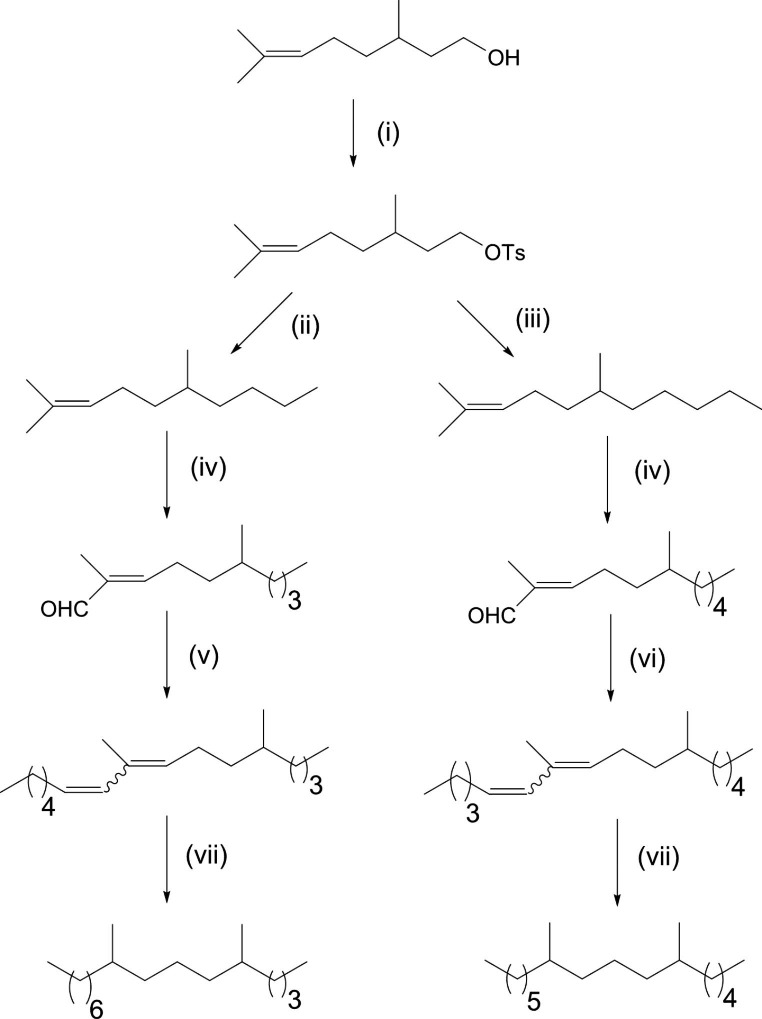
Synthesis of 6,10 dimethylhexadecane and 5,9 dimethylhexadecane. Reagents/conditions: (**i**) Tosyl chloride, pyridine; (**ii**) EtMgBr, Li_2_CuCl_2_, THF, -78 °C; (**iii**) PrMgBr, Li_2_CuCl_2_, THF, -78 C; (**iv**) tBuOOH, SeO_2_; (**v**) nBuLi, hexyltriphenyl phosphonium bromide, THF; (**vi**) nBuLi, pentyltriphenyl phosphonium bromide, THF; **vii**) Pd(C), H_2_, petroleum ether

Syntheses of 6,10 dimethyl hexadecane and 5,9 dimethylhexadecane were carried out according to the scheme in Fig. 2. Citronellol was tosylated in the presence of pyridine and the resulting tosylate was treated with ethyl magnesium bromide or propyl magnesium bromide in the presence of lithium tetrachlorocuprate to give the corresponding alkene intermediates. Oxidation of these alkenes with SeO_2_ gave a reasonable yield of the aldehydes, which were then subjected to the Wittig reaction giving the intermediate alkenes as mixtures of E and Z isomers. These mixtures were not separated prior to hydrogenation as the geometry of the double bonds was not relevant to the final product. Hydrogenation of the alkenes gave the dimethylhexadecanes as mixtures of diastereoisomers. Full details are provided in the Supplementary Material.

### Field Trapping Experiments

In two field trials conducted in this study, red delta traps (Clare et al. 2000) purchased from Etec Crop Solutions Ltd, Auckland, New Zealand were suspended at a height between 1 and 1.5 m on a metal wire running through a Scotch broom plantation adjacent to the Plant & Food Research Campus on Boundary Road, Lincoln, New Zealand (43°37’58.4"S; 172°28’57.9"E). Traps were deployed in a randomized block design, in which five trap lines were established at approx. 20-m intervals and five “trapping stations” were randomly assigned at approx. 20-m intervals in each trap line. In the first trial, 5-methylheptadecane (in 100 μl of *n*-hexane) was applied to a cotton ball placed inside a 2 ml clear screw top vial with a wide opening (Interlab Ltd, Wellington, New Zealand), the solvent was allowed to evaporate in the fume hood and vials were closed with lids and were stored in heat-sealed foil bags at − 20 °C until use. The lids were removed when the vials were placed inside the traps in the field. In the second trapping trial, the glass vial was compared to the white rubber septum (6 mm ID and 9 mm OD and 10 mm long, Sigma-Aldrich, St. Louis, MO, USA) as releasing substrate. 5-Methylheptadecane (in 100 μl of n-hexane) was added to the large ‘wells’ of white rubber septa. The solvent was allowed to evaporate in a fume hood and the vials and the septa were stored in heat-sealed foil bags at − 20°C until use. The pheromone-impregnated cotton ball inside the glass vial or septum was placed on the sticky panel of the trap. Traps baited with two virgin females 2–3 d old placed in a metal mesh cage (length × width = 3 × 2 cm) were used as a positive control. Females were changed each week and fed on a 10% sugar solution that was applied to a 1 cm dental roll placed inside the mesh cage. Traps baited with only the releasing substrate were used as a negative control.

In all field trials, five replicates for each treatment were tested. Traps were checked weekly, and moths were taken to the laboratory for identification and the total number of males per treatment was pooled for statistical analyses.

### Testing Various Doses of 5-Methylheptadecane

Three doses (0.01, 0.1, 1 mg) of 5-methylheptadecane loaded in a cotton ball in a glass vial were tested for catches of male *L. spartifoliella* in Scotch broom plantations in Lincoln, New Zealand. Traps baited with virgin females were used as positive controls, while traps baited with a cotton ball in a glass vial impregnated with only hexane were used as a negative control. The trial was deployed for four weeks from 15 October to 12 November 2021.

### Testing Cotton Ball in a Glass Vial vs. Rubber Septum

We tested two releasing substrates impregnated with 5-methylheptadecane for the attraction of male *L. spartifoliella* including: (a) white rubber septum; and (b) glass vial with a cotton ball. Each substrate was loaded with 1 mg 5-methylheptadecane. The trial was conducted in Scotch broom plantations in Lincoln, New Zealand. The trial was deployed for four weeks from 17 October to 14 November 2022.

### Data Analysis

The variances of mean captures of each treatment were normalized using √ (*x* + 1) transformation. In the first trial, the significance of differences between the different treatments was tested using ANOVA. Differences among means were tested using Fisher’s Protected Least Significant Difference (SAS Institute Inc. 1998). Treatments that caught no males were not included in the ANOVA since they showed no variance between replicates. In the second trial, the significance of differences in the mean total number of moths captured was tested using a paired *t*-test (SAS Institute Inc. 1998).

## Results

### Chemical Identification

**Fig. 3:**
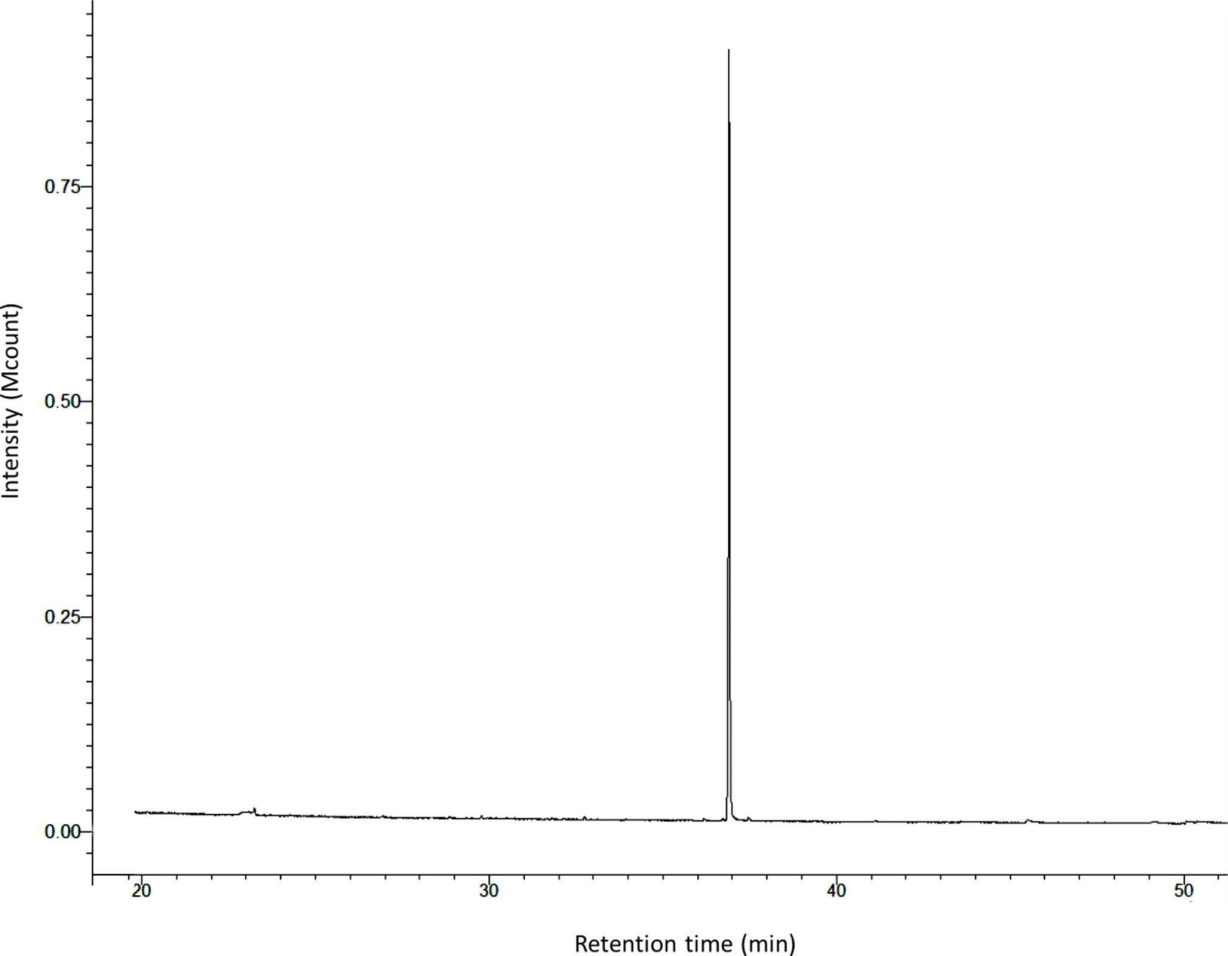
GC/MS total ion chromatogram of n-hexane extract of sex pheromone gland of female, *Leucoptera spartifoliella* showing only one peak on VF23-MS capillary column

**Fig. 4:**
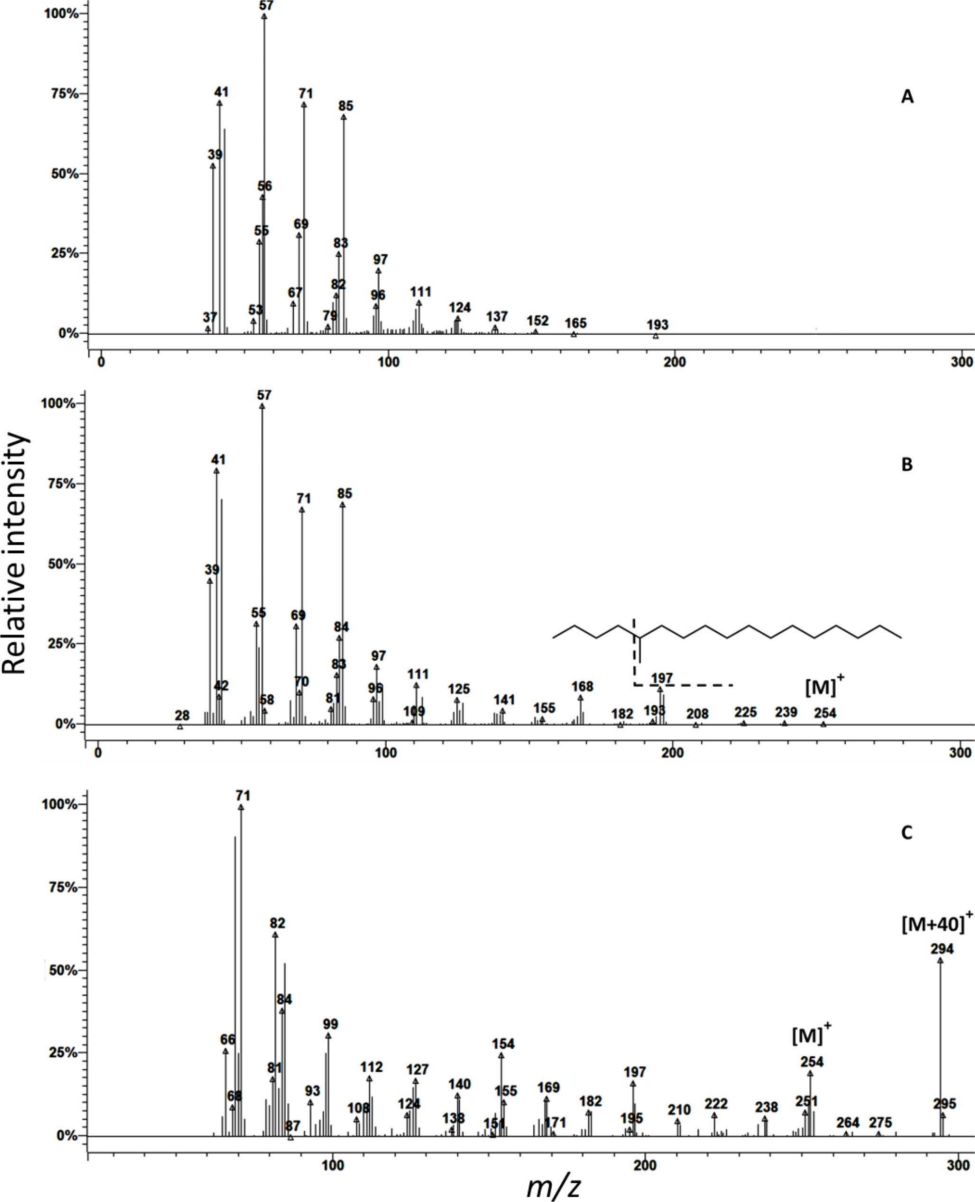
Mass spectra of the pheromone peak in the sex pheromone gland extract of female *Leucoptera spartifoliella* after electron ionization (EI) at (**A**) 250 °C, (**B**) 110 °C ion trap temperatures and (**C**) chemical ionization with acetonitrile

GC/MS analyses of gland extracts on the three different capillary columns indicated the presence of only one compound (Fig. 3). The retention indices of this compound were 1750 on a non-polar, VF5-MS capillary column, and 1742 on a polar VF23-MS capillary column which is in agreement with the published values of this compound (Köster et al. 1999). The mass spectral data of this compound showed a typical fragmentation pattern of hydrocarbon compounds, while its retention indices on a non-polar and polar capillary column suggested it was a branched hydrocarbon. At a higher ion-trap temperature (250 °C), no diagnostic ions were visible (Fig. 4A), while lowering the ion-trap temperature to 110 °C to allow for a softer fragmentation of molecule resulted in an appearance of several diagnostic ions such as *m/z* 168, *m/z* 197, and *m/z* 254 and enhancement of *m/z* 85 (Fig. 4B). Analysis of the gland extracts with chemical ionization with ACN (Fig. 4C) showed a significant diagnostic ion at *m/z* 294, which correspond to (M + C_2_H_2_N)^+^. This indicated that *m/z* 254 is the molecular ion of the female-produced compound, corresponding to a molecular formula of C_18_H_38_. Thus the main chain could be either 17 carbons with one methyl group or 16 carbons with two methyl groups or 15 carbons with three methyl groups.

However, with the retention indices of this compound 1750 on a non-polar column, this compound was unlikely to be 15 carbons with three methyl groups since a trimethylpentadecane would elute somewhere between 1580 and 1650 (Carlson et al. 1998). Similarly, a compound with 16 carbons with two methyl groups would be expected to elute somewhere between 1650 and 1710, and it was considered the compound was most likely to be a methyl-branched heptadecane. The diagnostic fragment at *m/z* 197 (M-57)^+^ and the enhancement of *m/z* 85 suggested 5-methylheptadecane or a dimethylhexadecane with one of the two methyl groups at position 5. On the other hand, the diagnostic fragment ion at *m/z* 168 suggested the possibility of dimethylhexadecane with one of the two methyl groups at position 10. Accordingly, we synthesised candidate compounds, 5-methylheptadecane, 5,9-dimethylhexadecane, and 6,10-dimethylhexadecane. The mass spectrum and retention indices of the synthetic 5-methylheptadecane exactly matched the main compound in the female gland extract. Analysis of the extract and synthetic 5-methylheptadecane on a chiral column did not resolve the stereoisomers of the compound produced by the female twig miner. The average amount (± SEM) of 5-methylheptadecane in the sex pheromone gland extracts was estimated to be 1.1 ± 0.24 ng/female.

### Field Testing Various Doses of 5-Methylheptadecane

**Fig. 5:**
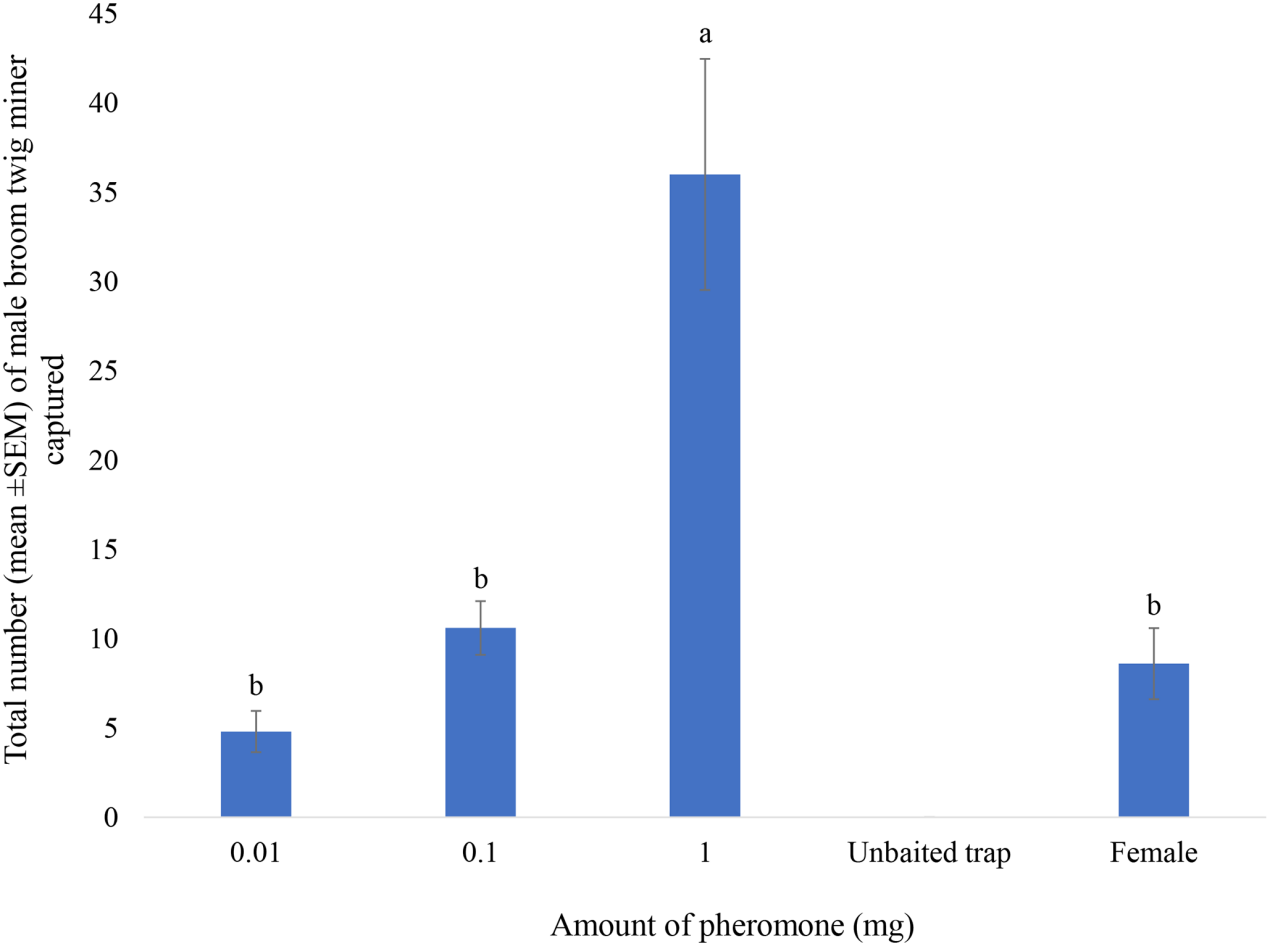
Total number (mean ± SEM) of male *Leucoptera spartifoliella* captured in traps baited with three doses (0.01, 0.1 and 1 mg) of 5-methylheptadecane and trap baited with virgin females. Different letters above columns indicate significant differences (*N* = 5, *P* < 0.05). Since no males were caught in the unbaited traps, they were not included in the ANOVA. The trial was deployed for 4 weeks from 15 October to 12 November 2021

The amount of 5-methylheptadecane loaded on the cotton ball inside the glass vial affected the number of male *L. spartifoliella* caught (*F*_3,16_ = 16.3, *P* < 0.01, Fig. 5). Increasing the dose from 0.01 to l mg resulted in a significant increase in the catch. The number of males caught in traps baited with 1 mg of synthetic 5-methylheptadecane was significantly higher than in traps baited with virgin females or 0.1 mg of 5-methylheptadecane. There was no difference between males caught in traps baited with virgin females or 0.1 mg 5-methylheptadecane (*P* < 0.01, Fig. 5). No males were caught in unbaited traps.

### Cotton Ball in a Glass Vial vs. Rubber Septum

The mean number of *L. spartifoliella* males captured in traps baited with 1 mg 5-methylheptadecane on cotton balls in glass vials (45.2 ± 5.1) was significantly different from the mean number (11.2 ± 1.6) of males captured in traps baited with 1 mg 5-methylheptadecane in white rubber septa (*t* = 7.6, df 4, *P* = 0.0016) over a four-week period.

## Discussion

In this study, the single compound 5-methylheptadecane has been identified as the sex pheromone of *L. spartifoliella*. The species belonging to the genus *Leucoptera*, are microlepdoptera and leaf miners, where some can cause severe damage to various plant crops. Up until now, the sex pheromone has been identified for only three species in the genus *Leucoptera*, this includes *L. coffeella* (Silvestri) (Francke et al. 1988), *L. malifoliella* (Costa) (Riba et al. 1990)d *sinuella* (Reutti) (Barros-Parada et al. 2020). In these three species, the sex pheromone has been identified as methyl-branched hydrocarbons (El-Sayed 2022). The identification of 5-methylheptadecane as a sex pheromone of *L. spartifoliella* is the fourth pheromone identification in this genus and is a methyl-branched hydrocarbon like the sex pheromones of the other three species in this genus. Contrary to the three *Leucoptera* species, where the main pheromone compound was dimethyl alkane, *L. spartifoliella* utilizes a mono-methyl alkane as a sex pheromone.

5-methylheptadecane has been identified as a trace compound in the sex pheromone gland in the female moths, *Pareuchaetes pseudoinsulata* (Régo Barros), which is a biological control agent of tropical bushes, *Chromolaena odorifera* (L.) (Schneider et al. 1992). Also, as a trace compound in the sex pheromone gland of the eastern and western hemlock looper, *Lambdina fiscellaria fiscellaria* (Guenée) and *Lambdina fiscellaria lugubrosa* (Hulst) (Gries et al. 1991; Gries 1993). In the three cases, 5-methylheptadecane did not affect trap catch positively or negatively. Therefore, this is the first case of pheromone identification, where 5-methylheptadecane has been identified as a major pheromone compound and biologically active in any moth.

Since there is one chiral carbon centre in 5-methylheptadecane, this compound can exist in two possible enantiomers. In this study, it was not possible to determine the absolute configuration of 5-methylheptadecane produced by females using a chiral column. Due to the lack of any functional group, the enantiomers of branched aliphatic hydrocarbons are very hard to be separated by chromatographic methods (Meierhenrich et al. 2003). For that reason, the absolute configurations of branched hydrocarbon sex pheromones are typically estimated by the field evaluation of synthetic stereoisomers. For example, the absolute configuration of the sex pheromone has not been determined for the other *Leucoptera* species using chromatographic analysis, instead, all possible enantiomers have been synthesised and tested for male attraction in field trapping trials. Toth et al. (1989) showed that male *L. malifoliella* were attracted only to (5* S*,9* S*)-5,9-dimethylheptadecane and the addition of any or all other enantiomers did not affect the number of males caught. In contrast, male *L. coffeella* responded equally to both (5*S*,9*R*)-dimethylpentadecane and the (5*S*,9*S*)-isomer, and both isomers caught a significantly greater number of males when compared with those captured by traps baited with the (5*R*,9*R*)-isomer, and (5*R*,9*S*)-isomer (Malo et al. 2009). In the first trapping trial described here, no significant difference was found between traps baited with virgin females and traps baited with 0.1 mg of racemic 5-methylheptadecane. This suggests that either female *L. spartifoliella* produce racemic 5-methylheptadecane or females produce one enantiomer, while the other enantiomer has no antagonistic effect. In addition to the Lepidopteran sex pheromones, some methyl-branched hydrocarbon pheromones have been identified from other insect orders. For example, 5,9,17-trimethylhenicosane has been identified as the sex pheromone of the true bug, *Phthia picta* (Drury) but its stereochemistry is unknown (Soldi et al. 2012).

In the trapping trial testing various doses of 5-methylheptadecane, the highest number of males were caught in traps baited with 1 mg loading. The number of males caught in 0.1 mg loading was similar to the number of males caught in traps baited with virgin females. In a subsequent trial testing cotton balls in glass vials and rubber septa loaded with 1 mg 5-methylheptadecane, the number of males caught in traps baited with a glass vial with a cotton ball was almost four times that in traps baited with rubber septa as releasing substrate over the four-week period. Unbranched alkanes will have lower volatility than branched alkanes, while mono-methyl alkanes will have lower volatility compared to dimethyl alkanes of the same carbon content. This is mainly because of the decrease in the surface areas which reduces the bonding force between molecules thus increasing the volatility as the structure change from unbranched, to mono-methyl and dimethyl alkanes. Thus when releasing unbranched alkanes or mono-methyl alkanes, a cotton ball/vial system would be a better substance because it offers low bonding force with low volatility molecules compared to rubber septa thus enhancing the release rate. Therefore, the observed difference between the two substrates could be attributed to a higher release rate of the cotton ball compared to the rubber septa. Accordingly, we recommend the use of a 1 mg dose loaded in a glass vial with a cotton ball for monitoring *L. spartifoliella*. Further work will be required to determine lure longevity, lure replacement and cost. The development of a monitoring system based on traps baited with the sex pheromone of *L. spartifoliella* can certainly help in the detection of newly released populations, determining population densities, distribution, and dispersal of this biocontrol agent.

## Electronic Supplementary Material

Below is the link to the electronic supplementary material.

Supplementary Material 1

## Data Availability

Data used in this study are available from the corresponding author upon request.

## Code Availability

(software application or custom code)

Not applicable.
