# Supplementary material for: 5-Methylheptadecane: Sex Pheromone of the Broom Twig Miner, Leucoptera Spartifoliella, a Biological Control Agent for the Scotch Broom
Source: J Chem Ecol. 2023 Jul 21;50(12):874–83. doi: 10.1007/s10886-023-01446-x (PMC11717844; doi:10.1007/s10886-023-01446-x)
Supplement: Supplementary file 1 — Supplementary Material 1 [file 10886_2023_1446_MOESM1_ESM.docx]

# Supplementary Material

# 5-Methylheptadecane: Sex Pheromone of the Broom Twig Miner, *Leucoptera spartifoliella,* a Biological Control Agent for the Scotch Broom

## Ashraf M. El-Sayed^1*^ and Barry Bunn^2^

### ^1^The New Zealand Institute for Plant and Food Research Limited, Canterbury Research Centre, Lincoln, 8152, New Zealand ^2^The New Zealand Institute for Plant and Food Research Limited, Batchelar Road, 4474, Palmerston North, New Zealand

^*^Author for correspondence email: ashraf.el-sayed@plantandfood.co.nz

# Synthesis of 6,10 Dimethylhexadecane and 5,9 Dimethylhexadecane

Syntheses of 6,10 dimethyl hexadecane and 5,9 dimethylhexadecane were carried out according to the scheme provided in Fig. 2. Citronellol was tosylated in the presence of pyridine and the resulting tosylate was treated with ethyl magnesium bromide or propyl magnesium bromide in the presence of lithium tetrachlorocuprate to give the corresponding alkene intermediates. Oxidation of these alkenes with SeO2 gave a reasonable yield of the aldehydes, which were then subjected to Wittig reaction giving the intermediate alkenes as mixtures of E and Z isomers. These mixtures were not separated prior to hydrogenation as the geometry of the double bonds was not relevant to the final product. Hydrogenation of the alkenes gave the dimethylhexadecanes as mixtures of diastereoisomers.

The model of NMR spectrometer used in this study was a Bruker Avance 500 and 700. For ^1^H the operating frequency was 500.13 MHz and 700.18MHz.

## Synthesis of 3,7-Dimethyloct-6-enyl 4-Methylbenzenesulfonate

Tosyl chloride (32.8 g, 172 mmol) was added portionwise over 30 min to citronellol (20 g, 128 mmol) in pyridine at 0 °C. The reaction was stirred at 0 °C for 1 h then allowed to warm to ambient temperature and stirred for 18 h. Water (15 ml) was added and the mixture stirred for 1 h. Water (160 ml) was added and the mixture was extracted with diethyl ether (3 x 200 ml). The combined organics were washed with NaHCO_3_ (1 x 100 ml, sat. aq.), brine (100 ml, sat. aq.) then dried over MgSO_4_. After filtration the solvent was removed *in vacuo*. The crude material was purified by column chromatography using 7% EtOAc / 93% petroleum ether as the eluant. Yield = 31.9 g, 81%. NMR data were in agreement with the NMR data reported by Doan et al. (2007).

## Synthesis of 2,6-Dimethyldec-2-ene

Part 1. A solution of lithium tetrachlorocuprate was prepared as follows. Dry THF (6 ml) was added under N_2_ to LiCl (39 mg, 0.93 mmol) and anhydrous CuCl_2_ (60 mg, 0.45 mmol). Part 2. Ethylmagnesium bromide (16.6 ml, 49.8 mmol, 3.0 M in Et_2_O) was added dropwise over 5 min under N_2_ to a solution of 3,7-dimethyloct-6-enyl 4-methylbenzenesulfonate (10.3 g, 33 mmol) in THF (120 ml) at −78 °C. To this mixture was then added dropwise over 1 min 2.6 ml of the Li_2_CuCl_2_ solution from part 1. The reaction was stirred at −78 °C for 30 min then allowed to warm to ambient temperature and stirred for 18 h. NH_4_Cl (50 ml, sat. aq.) was added and the mixture was extracted with diethyl ether (2 x 100 ml). The combined organics were washed with brine (50 ml, sat. aq.) then dried over MgSO_4_. After filtration, the solvent was removed *in vacuo*. The crude material was purified by column chromatography using 5% EtOAc / 95% petroleum ether as eluant. Yield = 3.1 g, 56%. NMR data were in agreement with the NMR data reported by Liang et al. (2000).

## Synthesis of (*E*)-2,6-Dimethyldec-2-enal

Salicylic acid (23 mg, 0.16 mmol) and SeO_2_ (15 mg, 0.14 mmol) were stirred in dichoromethane (4 ml) at ambient temperature. A solution of *tert* butylhydroperoxide (0.8 ml) in dichloromethane (1.8 ml) was then added. The solution was cooled to 0 °C and stirred for 10 min at this temperature. A solution of 2,6-dimethyldec-2-ene (354 mg, 2.3 mmol), in dichloromethane (4 ml) was then added dropwise over 1 min. The reaction was allowed to warm to ambient temperature and stirred for 6 h at which point more SeO_2_ (20 mg, 0.19 mmol) was added. The reaction was stirred for 18 h at ambient temperature. Water (10 ml) was added followed by FeSO_4_ (30 mg, 0.2 mmol) and the reaction stirred for 10 min. The mixture was extracted with dichloromethane (3 x 20 ml) then the combined organics washed with brine (10 ml, sat. aq.), dried over MgSO_4_ and the solvent removed *in vacuo*. The crude material was purified by column chromatography using 5% EtOAc / 95% petroleum ether as eluant. Yield = 85 mg, 22%. NMR data were in agreement with NMR data reported by Liang et al. (2000).

## Synthesis of (6,8)-8,12-Dimethylhexadeca-6,8-diene

^n^Butyl lithium (0.18 ml, 0.36 mmol, 2.0 M in cyclohexane) was added dropwise under N_2_ over 1 min to a suspension of hexyltriphenylphosphonium bromide (131 mg, 0.31 mmol) in THF (5 ml) at −20 °C. An intense orange colour was observed. This mixture was stirred at −20 °C for 1 h at which point (*E*)-2,6-dimethyldec-2-enal (62 mg, 34 mmol) was added dropwise over 2 min as a solution in THF (1 ml). The reaction was allowed to warm to ambient temperature and stirred for 18 h. NH_4_Cl (5 ml, sat. aq.) was added and the mixture was extracted with diethyl ether (3 x 10 ml). The combined organics were washed with brine (5 ml, sat. aq.) then dried over MgSO_4_. After filtration, the solvent was removed *in vacuo*. The crude material was purified by column chromatography using 3% EtOAc / 97% petroleum ether as eluant to give the product as a mixture of *E* and *Z* isomers as determined by GC/MS. Yield = 22 mg, 24%.

## Synthesis of 5,9-Dimethylhexadecane

(6,8)-8,12-Dimethylhexadeca-6,8-diene (a mixture of 6*E* and 6*Z* isomers) (20 mg, 0.08 mmol) in petroleum ether (30 ml) was hydrogenated at ambient temperature in the presence of 10% palladium on carbon (10 mg) for 1 h. The catalyst was removed by filtration through celite and the solvent was removed in vacuo. The product was obtained as a mixture of diastereoisomers. Yield = 13 mg, 64%. NMR data were in agreement with NMR data reported by Liang et al. (2000).

## Synthesis of 2,6-Dimethylundec-2-ene

Part 1. A solution of lithium tetrachlococuprate was prepared as follows. Dry THF (6 ml) was added to LiCl (39 mg, 0.93 mmol) and anhydrous CuCl_2_ (60 mg, 0.45 mmol) under N_2_. Part 2. Propylmagnesium bromide (22.3 ml, 44.5 mmol, 2.0 M in Et_2_O) was added dropwise over 10 min to a solution of 3,7-dimethyloct-6-enyl 4-methylbenzenesulfonate (9.2 g, 29.6 mmol) in THF (120 ml) under N_2_ at −78 °C. To this mixture was then added dropwise over 1 min 2.4 ml of the Li_2_CuCl_2_ solution from part 1. There was an immediate orange colour. The reaction was stirred at −78 °C for 30 min then allowed to warm to ambient temperature and stirred for 18 h. NH_4_Cl (50 ml, sat. aq.) was added and the mixture was extracted with diethyl ether (2 x 100 ml). The combined organics were washed with brine (50 ml, sat. aq.) then dried over MgSO_4_. After filtration, the solvent was removed *in vacuo*. The crude material was purified by column chromatography using 5% EtOAc / 95% petroleum ether as eluant. Yield = 3.1 g, 61%. NMR data are in agreement with the NMR data reported by Matt et al. (2020).

## Synthesis of (*E*)-2,6-Dimethylundec-2-enal

Part 1. *tert* Butylhydroperoxide (50 ml, 70% in H_2_O) was added over 10 min to dichloromethane (100 ml). After drying over MgSO_4_, the solids were removed by decantation. Part 2. Salicylic acid (190 mg, 1.4 mmol) and SeO_2_ (120 mg, 1.1 mmol) were stirred in dichloromethane (10 ml) at ambient temperature. A solution of *tert-*butylhydroperoxide in dichloromethane from part 1 (18 ml) was then added dropwise over 5 min. The solution was cooled to 0 °C and stirred for 5 min at this temperature. A solution of 2,6-dimethylundec-2-ene (2.97 g, 16.3 mmol), in dichloromethane (10 ml) was then added dropwise over 10 min. The reaction was allowed to warm to ambient temperature and stirred for 6 h at which point SeO_2_ (150 mg, 1.4 mmol) was added. The reaction was stirred for 18 h at ambient temperature. Water (10 ml) was added followed by FeSO_4_ (150 mg, 1 mmol) and the reaction was stirred for 10 min. The mixture was extracted with dichloromethane (3 x 30 ml) then the combined organics were washed with brine (20 ml, sat. aq.), dried over MgSO_4_ and then the solvent was removed *in vacuo*. The crude material was purified by column chromatography using 5% EtOAc / 95% petroleum ether as eluant. Yield = 1.30 g, 41%. ^1^H NMR (500 Mhz. CDCl_3_): δ 9.42 (1 H, s) 6.51 (tr d *J* = 7.5, 1 Hz) 2.3 (2 H, m) 1.77 (3 H, s) 1.50 (2 H, m) 1.39–1.26 (8 H, m) 0.94 (3 H, tr, *J* = 7.5 Hz) 0.93 (3 H, tr, *J* = 7.5 Hz); ^13^C NMR (125 MHz. CDCl_3_) δ 195.4, 155.4, 155.3, 36.8, 35.6, 35.5, 32.6, 32.5, 32.2, 26.7, 22.7, 19.5, 14.1.

## Synthesis of (5,7)-7,11-Dimethylhexadeca-5,7-diene (mixture of 5*E* and 5*Z* isomers)

^n^Butyl lithium (1.5 ml, 2.4 mmol, 2.0 M in cyclohexane) was added dropwise over 3 min to a suspension of pentyltriphenyl phosphonium bromide (1.02 g, 2.4 mmol) in THF (30 ml) at −20 °C. An immediate orange colour was observed. This mixture was stirred at −20 °C for 1 h when (*E*)-2,6-dimethylundec-2-enal (590 mg, 3 mmol) in THF (5 ml) was added dropwise over 5 min. The reaction was then allowed to warm to ambient temperature and stirred for 18 h. NH4Cl (20 ml, sat. aq.) was added and the aqueous phase was extracted with diethyl ether (2 x 30 ml), the combined organics were washed with brine (20 ml, sat. aq.) and then dried over MgSO_4_. After filtration, the solvent was removed *in vacuo*. The crude material was purified by column chromatography using 3% EtOAc / 97 petroleum ether as an eluant. The 5*E* and 5*Z* isomers could not be separated by column chromatography and were used as a mixture in the next step. Yield = 98 mg (mixture of *E* and *Z* isomers in the ratio 3.5:1), 10%.

## Synthesis of 6,10 Dimethylhexadecane

(5,7)-7,11-Dimethylhexadeca-5,7-diene (25 mg, 0.1 mmol) (mixture of 5*E* and 5*Z* isomers) in petroleum ether (30 ml) was hydrogenated at ambient temperature in the presence of 10% palladium on carbon (10 mg) for 1 h. The catalyst was removed by filtration through celite and the solvent was removed *in vacuo*. The product was obtained as a mixture of diastereoisomers. Yield = 11 mg, 43%. ^1^H NMR (500 Mhz. CDCl_3_) δ 0.87 (6 H, 2 x d overlapping, *J* = 6.3 Hz), 0.91 (6 H, 2 x tr overlapping, *J* = 7.8 Hz), 1.25–1.40 (24H, br). ^13^C NMR (125MHz. CDCl_3_) δ 37.5, 37.4, 37.2, 37.1, 37.0, 37.0, 32.8, 32.7, 32.3, 32.0, 29.7, 27.1, 26.8, 24.5, 22.8, 22.7, 19.8, 19.7, 14.1.

# References

Doan N, Le T, Nguyen ,H, Hansen P, Duus F (2007) Ultrasound assisted synthesis of 5,9-dimethylpentadecane and 5,9-dimethylhexadecane-the sex pheromones of *Leucoptera coffeella*. Molecules 12:2080-2088

Liang T, Kuwahara S, Hasegawa M, Kodama O (2000) Simple synthesis of 5,9-dimethylated long-chain alkanes, the sex pheromones of leaf miner moths. Biosci Biotechnol Biochem 64:2474-2477.

Matt C, Kern, C, Streuff J (2020) Zirconium-catalyzed remote defunctionalization of alkenes. ACS Catal 10:6409-6413
